# Compression Test for Diagnosis of Phalangeal Fracture; a Letter to Editor

**Published:** 2019-09-15

**Authors:** Naser Mohamad Karimi, Faeze Zeinali.N

**Affiliations:** 1 Department of Emergency Medicine, Shahid Sadoughi University of Medical Sciences, Yazd, Iran.

**Keywords:** Fractures, compression, clinical decision-making, physical examination, diagnostic imaging, radiography

Physical examination is the most important procedure for examining traumatized patients and ruling out possible bone fractures. Edema, deformity, ecchymosis, tenderness on trauma location, and limited bone movement are among the signs of fracture. The diagnosis is documented by plain radiography. The number of false positive cases in this test increase in the presence of soft tissue injury along with bone damage. 

In this case, a great number of X-rays will be taken daily and many patients will be exposed to radiation, unnecessarily (1). The question to be fully answered is whether all cases of trauma and pain require radiographs or not? 

In cases of suspicion to scaphoid bone fractures, scaphoid compression (compressing a patient’s thumb along the line of the first metacarpal bone) is used as a clinical test. This test is said to have sensitivity of 70.5%, specificity of 21.8%, and predictive value of 41.9% for diagnosing scaphoid bone fracture (2). Given the similarities present in force transmission and creation of pain in both scaphoid bone and phalanges, we assessed the mentioned maneuver in a similar fashion for phalanges and investigated the value of “axially/longitudinally compressing a patient’s finger along the longitudinal line of phalanxes” as a test for predicting phalangeal bone fracture in patients with traumatized phalanges. 

In this study, 100 patients with direct blunt trauma to hand were examined. The mean age of patients was 31.18 ± 11.05 years (85% male). All patients underwent compression test and radiography. One emergency medicine physician and one radiologist interpreted the radiographies, separately. The total sensitivity and specificity of test were 0.673 (95% CI: 0.485 – 0.895) and 0.875 (95% CI: 0.987 – 0.692), respectively. Sensitivity and specificity of test for diagnosis of proximal phalanx fracture were 100% and 100%, respectively. 

Our findings demonstrated that in the presence of fracture involving the whole width of phalange of each finger, the test will be positive. Hence, this test enjoys a high predictive value for diagnosing bone fractures and it can be used to reduce the need for X-rays ordered for phalanges. Of course, some exceptions ought to be clarified. Regarding joints, 26 victims had pain and tenderness on phalangeal joints. However, only 5 of them felt severe pain on scaphoid compression test, while, interestingly, none of them had fractures on radiography. On the other hand, out of the 21 victims that had no pain during the examination, 3 had avulsion fracture (FX) in the radiography, which also involved the joint surface, slightly. This may be attributed to the point that this type of fracture does not involve all the width of the phalanx and is not located on the longitudinal axis of the bone and in the path of force transmission, so the patient does not feel any pain in this region. 

On the whole, it may be concluded that considering the presence of various types of soft tissues in the joint, such as joint capsule, synovial fluid, and tendons in the joint space, force transmission is not done completely; hence, this test is not of much diagnostic value in diagnosing joint damage. 
